# Supplementary material for: Comparative regulomics supports pervasive selection on gene dosage following whole genome duplication
Source: Genome Biol. 2021 Apr 13;22:103. doi: 10.1186/s13059-021-02323-0 (PMC8042706; doi:10.1186/s13059-021-02323-0)
Supplement: Supplementary file 2 — Additional file 2. Review history. [file 13059_2021_2323_MOESM2_ESM.docx]

Review History

**First round of review**

**Reviewer 1**

**Are you able to assess all statistics in the manuscript, including the appropriateness of statistical tests used?**

Yes, and I have assessed the statistics in my report.

**Comments to author:**

Review of Gillard et al., "Comparative regulomics reveals pervasive selection on gene dosage following whole genome duplication."

Overview:

In this manuscript, the authors explore how gene expression has changed genome-wide in the polyploidy salmonids, using outgroup diploids as a reference. Using a phylogenetic model of expression evolution, they find that the predominant mode of expression evolution is either an asymmetric or symmetric decrease in expression level among surviving duplicates (ohnologs), presumably to preserve dosage balance with other genes not surviving in duplicate from the polyploidy. Mechanisms for these changes can be detected in the loss of transcription factor binding sites for the more lowly expressed copies of the genes. The authors also detect rarer cases of potentially adaptive changes in expression for a few of the surviving duplicates.

Major comments:

This is a very compelling manuscript on a key question regarding evolution after polyploidy. The strength of the paper is in the careful evolutionary modeling of expression evolution using the UR model coupled to measured expression levels in multiple polyploidy and diploid taxa. While there is other literature on expression evolution in polyploids, this paper is the first (to my knowledge) to use such models. This is a critical improvement, because the prior work was not rigorous in distinguishing the actions of selection from those of drift in assessing evolutionary changes in expression level. Hence, this paper is a key step forward in this area.

I also find the paper to be overall quite clear and straightforward to read. My comments are mostly focused on some relatively minor issues of interpretation.

At the top of page 6: the definition of biased fractionation given is a bit different than that I tend to read elsewhere. In other work, biased fractionation refers to a pattern of ohnolog losses that are biased against one of the allopolyploid parents. The authors use the term also in this sense, but what they are discussing here might be more accurately described as a pattern of differential selection acting on ohnologs that could drive biased fractionation. (Also note that this is not the only proposed origin of biased fractionation).

On page 6: A deficit of ohnologs among genes targeted to organelles has been observed for plants 1, and I suspect the OxPhos results described here may be a similar phenomenon. One argument made in this context is that a polyploidy event usually will not change the mitochondrial genome dosage, meaning there is a potential for imbalance between the nuclear and mitochondrial genomes, potentially resulting in selection for ohnolog loss or expression reduction in the nuclear-encoded genes whose products are targeted to the mitochondria.

Ribosomal proteins are often observed to be over-retained after polyploidy:2 I wonder whether in the alternative case where some of the ohnologs are lost there might be particularly strong selection on reducing dosage in others.

In this vein, I think the authors discussion of absolute and relative dosage on pages 6&7 might benefit from some further elaboration. The dosage balance hypothesis can be described in terms of protein complexes, but "interaction" in this context could equally well be regulatory or functional. Hence, I think the dismissal of "relative dosage" here might be a bit too strong. Another test that could be run is to ask if singletons and ohnologs co-occur in complexes more rarely than expected. Also, "absolute dosage" has been used in a few different ways: in particular, to mean selection to tune, say, the overall protein abundance of enzymes for a metabolic pathway for increased flux. I think the authors' use differs from this one, because they are still arguing about how expression of ohnologs is changing to be "in sync" with singletons, which to my mind, is something closer to "relative dosage."

Page 7, lines 10 and following: I was not clear on whether the asymmetry in expression evolution was being compared to that in sequence evolution, or if the analysis was something else again.

Page 4, line 26 "it's" "its"

References:

1. De Smet, R. et al. Convergent gene loss following gene and genome duplications creates single-copy families in flowering plants. Proceedings of the National Academy of Sciences, U.S.A. 110, 2898-903 (2013).

2. Blanc, G. & Wolfe, K.H. Functional divergence of duplicated genes formed by polyploidy during Arabidopsis evolution. Plant Cell 16, 1679-1691 (2004).

**Reviewer 2**

**Are you able to assess all statistics in the manuscript, including the appropriateness of statistical tests used?**

No, I do not feel adequately qualified to assess the statistics.

**Comments to author:**

The authors of this manuscript focused on whole genome duplication in the Salmonidae lineage and investigated the effect of selection on gene expression after the whole genome duplication. Overall, the study is not designed on a firm ground of evolutionary theories or understanding in transcriptional regulation of gene expression, as reflected in ambiguous expressions in the manuscript, such as 'ohnolog expression levels have evolved', 'gene expression phenotypes', 'gene expression evolution', 'for becoming a successful polyploid lineage', and 'transcription factor binding site evolution'. The use of the 'phylogenetic OU process model', which is not well explained in the manuscript, does not warrant a priori the validity of the authors' approach in this line of data presentation. Moreover, transcriptional profiling alone does not instruct us with adaptive effects of gene functions on phenotypes, even combined with an analysis on transcription factor binding landscape. Critically, the question to be addressed, the methods applied, and the conclusion gained are not carefully described, which makes this manuscript difficult to follow, especially for a wide range of readers of this journal. The abstract also lacks the details of their methods and concrete findings.

Page 12, line 22 'the same genes and pathways' - should not be the same if across different species, phyla, or even kingdoms.

'Ss4R' should be explained at least once in the main text.

**Authors Response**

**Point-by-point responses to the reviewers’ comments:**

Reviewer #1: Review of Gillard et al., "Comparative regulomics reveals pervasive selection on gene dosage following whole genome duplication."

Overview:

In this manuscript, the authors explore how gene expression has changed genome-wide in the polyploidy salmonids, using outgroup diploids as a reference. Using a phylogenetic model of expression evolution, they find that the predominant mode of expression evolution is either an asymmetric or symmetric decrease in expression level among surviving duplicates (ohnologs), presumably to preserve dosage balance with other genes not surviving in duplicate from the polyploidy. Mechanisms for these changes can be detected in the loss of transcription factor binding sites for the more lowly expressed copies of the genes. The authors also detect rarer cases of potentially adaptive changes in expression for a few of the surviving duplicates.

Major comments:

This is a very compelling manuscript on a key question regarding evolution after polyploidy. The strength of the paper is in the careful evolutionary modeling of expression evolution using the UR model coupled to measured expression levels in multiple polyploidy and diploid taxa. While there is other literature on expression evolution in polyploids, this paper is the first (to my knowledge) to use such models. This is a critical improvement, because the prior work was not rigorous in distinguishing the actions of selection from those of drift in assessing evolutionary changes in expression level. Hence, this paper is a key step forward in this area.

I also find the paper to be overall quite clear and straightforward to read. My comments are mostly focused on some relatively minor issues of interpretation.

*Answer: Thank you for a thorough and positive review. Our point-by-point responses are given below.*

At the top of page 6: the definition of biased fractionation given is a bit different than that I tend to read elsewhere. In other work, biased fractionation refers to a pattern of ohnolog losses that are biased against one of the allopolyploid parents. The authors use the term also in this sense, but what they are discussing here might be more accurately described as a pattern of differential selection acting on ohnologs that could drive biased fractionation. (Also note that this is not the only proposed origin of biased fractionation).

*Answer: This is a good point. We have rewritten the paragraph on biased fractionation with these comments in mind:*

*«Pervasive differences in purifying selection pressure within individual ohnolog pairs raise the question of whether these ohnologs might belong to duplicated genome blocks experiencing large-scale differences in selective constraints. This could lead to uneven ohnolog loss rates, a process that is referred to as biased fractionation [1]. In line with previous studies on teleosts (Conant 2019; Xu et al. 2019) we found significant biases in local gene loss, albeit only in 9 of 47 syntenic duplicate blocks. However, we did not find equivalent large-scale biases in expression loss (Supplementary figure 9), thus rendering regional differences in selection constraints an unlikely explanation for the large number of ohnologs experiencing loss of expression in one copy.»*

On page 6: A deficit of ohnologs among genes targeted to organelles has been observed for plants 1, and I suspect the OxPhos results described here may be a similar phenomenon. One argument made in this context is that a polyploidy event usually will not change the mitochondrial genome dosage, meaning there is a potential for imbalance between the nuclear and mitochondrial genomes, potentially resulting in selection for ohnolog loss or expression reduction in the nuclear-encoded genes whose products are targeted to the mitochondria.

*Answer: This is again a very good point. We have rewritten the second section of the Discussion (“Long term ohnolog retention and selection on gene dosage”) with this interpretation in mind. We have also added the results from testing if “singletons and ohnologs co-occur in complexes more rarely than expected”, which the reviewer suggests further down.*

*Revised text in Discussion:*

*“Following an early phase of selection on gene dosage, the long term fates of ohnologs can be shaped by various adaptive processes [2, 3], including adaptive regulatory evolution. One potential outcome is adaptive divergence between ohnologs, resulting in two functionally non-redundant ohnologs under purifying selection. Our results demonstrate that tissue-specific shifts (up+cons) in expression are rare (Figure 1C), and interpret this to mean that adaptive evolution of novel tissue-specific regulation likely has very little impact on genome wide ohnolog retention.*

*Selection on molecular stoichiometry has been proposed to play a major role in genome evolution after WGDs [4]. This narrative is supported by our finding that molecular complexes are both enriched for retained ohnologs and biased to include only singletons or only ohnologs (Supplementary figure 14). However, selection on molecular stoichiometry could also drive evolution of gene expression. Indeed, we do find some (but weak) support for selection on stoichiometric balance also operating through selection for higher expression levels of singleton genes that are in complexes with ohnologs (Supplementary table 3, p=0.07). Moreover, it is plausible that the strong bias of ‘oxidative phosphorylation’-ohnologs towards highly asymmetric expression regulation also is linked to selection on stoichiometry (Figure 2B-C). These genes are nuclear encoded genes involved in energy-related functions in mitochondria. As WGDs do not double the plastid numbers it has been proposed that in plants stoichiometric imbalances between nuclear and plastid genomes act as selection pressure to reduce the ratio of nuclear to plastid gene dosage following WGD [5]. In line with this reasoning, the driver behind the strong asymmetric down shift of ‘oxidative phosphorylation’-ohnologs could be the reinstatement of stoichiometric balance between the nuclear and mitochondrial genes.*

*At the other end of the ohnolog expression evolution symmetry spectrum, we find ohnologs belonging to the ‘ribosomal protein’ pathway evolving lower expression in a highly symmetric fashion (Figure 2B-C). We also demonstrate a significant correlation between constraints at the coding sequence level and symmetry of ohnolog regulatory evolution (Figure 2D). This is in line with findings from plants that ribosomal proteins are retained over long evolutionary times and evolve slowly at both sequence and expression levels following WGD [6]. One potential explanation for this pattern could be the ‘toxic effects model’ where long term conservation of ohnologs is intrinsically linked to the ‘danger’ of accumulating highly toxic coding sequence mutations [7, 8]. We therefore hypothesize that in situations where lowering the total gene dosage increases fitness, and the tolerance for accumulation of deleterious mutations is low (i.e. the toxic effect), symmetric ohnolog evolution towards lower gene dosage could be favoured over pseudogenization of one copy”*

Ribosomal proteins are often observed to be over-retained after polyploidy:2 I wonder whether in the alternative case where some of the ohnologs are lost there might be particularly strong selection on reducing dosage in others. In this vein, I think the authors discussion of absolute and relative dosage on pages 6&7 might benefit from some further elaboration. The dosage balance hypothesis can be described in terms of protein complexes, but "interaction" in this context could equally well be regulatory or functional. Hence, I think the dismissal of "relative dosage" here might be a bit too strong. Another test that could be run is to ask if singletons and ohnologs co-occur in complexes more rarely than expected.

Also, "absolute dosage" has been used in a few different ways: in particular, to mean selection to tune, say, the overall protein abundance of enzymes for a metabolic pathway for increased flux. I think the authors' use differs from this one, because they are still arguing about how expression of ohnologs is changing to be "in sync" with singletons, which to my mind, is something closer to "relative dosage."

*Answer:*

*We agree that our original text/interpretation was too strong regarding the importance of relative dosage selection and that our use of ‘absolute dosage’ in the conclusion section was misleading. We have thus rephrased and provided (hopefully) a more nuanced/balanced text regarding gene dosage selection.*

*Revised text in the Results section:*

*“Taken together, we find strong evidence for dosage selection following WGD on genes involved in basic cellular maintenance and cell division. In addition, we find evidence for selection to retain stoichiometric balance both at the sequence and expression level.”*

*Revised text in the Conclusion section:*

*“Since gene loss is absent in early generations polyploids, and all genes are duplicated and in stoichiometric balance, early plastic changes in gene regulatory phenotypes is likely a result of deleterious fitness effects due to suboptimal absolute gene dosages.”*

*We have also performed the additional analysis as suggested by the reviewer, and reported the findings in the results. Also see our changes to the second section of the Discussion.*

Page 7, lines 10 and following: I was not clear on whether the asymmetry in expression evolution was being compared to that in sequence evolution, or if the analysis was something else again.

*Answer: We believe the confusion could be due to two paragraphs unintentionally having been merged together. We have corrected this and revised the text slightly to enhance clarity.*

Page 4, line 26 "it's" "its"

*Answer: Corrected.*

Reviewer #2: The authors of this manuscript focused on whole genome duplication in the Salmonidae lineage and investigated the effect of selection on gene expression after the whole genome duplication. Overall, the study is not designed on a firm ground of evolutionary theories or understanding in transcriptional regulation of gene expression, as reflected in ambiguous expressions in the manuscript, such as 'ohnolog expression levels have evolved', 'gene expression phenotypes', 'gene expression evolution', 'for becoming a successful polyploid lineage', and 'transcription factor binding site evolution'. The use of the 'phylogenetic OU process model', which is not well explained in the manuscript, does not warrant a priori the validity of the authors' approach in this line of data presentation. Moreover, transcriptional profiling alone does not instruct us with adaptive effects of gene functions on phenotypes, even combined with an analysis on transcription factor binding landscape. Critically, the question to be addressed, the methods applied, and the conclusion gained are not carefully described, which makes this manuscript difficult to follow, especially for a wide range of readers of this journal. The abstract also lacks the details of their methods and concrete findings.

*Answer:*

*The reviewer raises a number of concerns without explanation or references to specific analysis or text, which makes them hard to address. We have gone through the manuscript and rephrased some sentences where the mentioned expressions are used - based on our best guess of what it is that the reviewer objects to, including revising “gene expression phenotypes” to “gene expression levels”. We have also reworked the abstract with the reviewer’s comments in mind.*

*The OU method is described in some detail in the supplementary material and Methods section, and of course in mathematical detail in the original method papers ([9, 10]). Genome Biology’s formatting rules do not allow for a method section in the abstract.*

Page 12, line 22 'the same genes and pathways' - should not be the same if across different species, phyla, or even kingdoms.

*Answer: Changed to homologous.*

'Ss4R' should be explained at least once in the main text.

*Answer: Corrected.*

*References*

*1. Langham RJ, Walsh J, Dunn M, Ko C, Goff SA, Freeling M. Genomic duplication, fractionation and the origin of regulatory novelty. Genetics. 2004;166:935–45.*

*2. Conant GC, Wolfe KH. Turning a hobby into a job: how duplicated genes find new functions. Nat Rev Genet. 2008;9:938–50. doi:10.1038/nrg2482.*

*3. Hallin J, Landry CR. Regulation plays a multifaceted role in the retention of gene duplicates. PLoS Biol. 2019;17:e3000519. doi:10.1371/journal.pbio.3000519.*

*4. Birchler JA, Veitia RA. Gene balance hypothesis: connecting issues of dosage sensitivity across biological disciplines. Proc Natl Acad Sci USA. 2012;109:14746–53. doi:10.1073/pnas.1207726109.*

*5. De Smet R, Adams KL, Vandepoele K, Van Montagu MCE, Maere S, Van de Peer Y. Convergent gene loss following gene and genome duplications creates single-copy families in flowering plants. Proc Natl Acad Sci USA. 2013;110:2898–903. doi:10.1073/pnas.1300127110.*

*6. Blanc G, Wolfe KH. Functional divergence of duplicated genes formed by polyploidy during Arabidopsis evolution. Plant Cell. 2004;16:1679–91. doi:10.1105/tpc.021410.*

*7. Roux J, Liu J, Robinson-Rechavi M. Selective constraints on coding sequences of nervous system genes are a major determinant of duplicate gene retention in vertebrates. Mol Biol Evol. 2017;34:2773–91. doi:10.1093/molbev/msx199.*

*8. Singh PP, Affeldt S, Cascone I, Selimoglu R, Camonis J, Isambert H. On the expansion of “dangerous” gene repertoires by whole-genome duplications in early vertebrates. Cell Rep. 2012;2:1387–98. doi:10.1016/j.celrep.2012.09.034.*

*9. Rohlfs RV, Nielsen R. Phylogenetic ANOVA: the expression variance and evolution model for quantitative trait evolution. Syst Biol. 2015;64:695–708. doi:10.1093/sysbio/syv042.*

*10. Rohlfs RV, Harrigan P, Nielsen R. Modeling gene expression evolution with an extended Ornstein-Uhlenbeck process accounting for within-species variation. Mol Biol Evol. 2014;31:201–11. doi:10.1093/molbev/mst190.*

**Second round of review**

**Reviewer 1**

Review of Gillard et al., "Comparative regulomics supports pervasive selection on gene dosage following whole genome duplication."

Overview:

The authors explore how gene expression has changed genome-wide in the polyploidy salmonids, using outgroup diploids as a reference. Using a phylogenetic model of expression evolution, they find that the predominant mode of expression evolution is either an asymmetric or symmetric decrease in expression level among surviving duplicates (ohnologs), presumably to preserve dosage balance with other genes not surviving in duplicate from the polyploidy. Mechanisms for these changes can be detected in the loss of transcription factor binding sites for the more lowly expressed copies of the genes. The authors also detect rarer cases of potentially adaptive changes in expression for a few of the surviving duplicates.

Major comments:

The authors have addressed my major concerns from the previous submission, and I have no serious concerns with the manuscript as it stands. Some more minor suggestions:

Pg 2, Line 23: possibly -> possible

Pg 3, Line 42: "WGD leads to" I think the general statement here is a bit strong: the authors have a particular finding about the Ss4R event. Maybe "We find that this WGD led to…"

Pg 5, Line 58: "lowest expressed copy" -> "more lowly expressed copy"

Pg 6, Line 49:It is not clear to me whether dosage balance, as distinct from genomic integrity, is an alternative hypothesis for these results.

Pg 12, Line 31: The toxic effects model is very relevant here, but the authors should also consult (De Smet et al. 2013), which proposes the opposite effect: selection to remove ohnolog copies to avoid dominant negative mutations.
